# A194 THE ADDITION OF DEAMIDATED GLIADIN PEPTIDE TO TISSUE TRANSGLUTAMINASE ANTIBODIES DOES NOT INCREASE THE ODDS OF CELIAC DISEASE DIAGNOSIS IN AN IGA SUFFICIENT POPULATION

**DOI:** 10.1093/jcag/gwab049.193

**Published:** 2022-02-21

**Authors:** K Moss, L Russell, K Mehta, M Faisal, D Armstrong, E Verdu, J Dowhaniuk, M I Pinto-Sanchez

**Affiliations:** McMaster University Faculty of Health Sciences, Hamilton, ON, Canada

## Abstract

**Background:**

Previous studies proposed that the combination of IgA anti-tissue transglutaminase 2 IgA (TTG) and IgG deamidated gliadin peptide IgG (DGP) antibodies increases celiac disease (CeD) detection rates. However, this remains controversial.

**Aims:**

To evaluate the performance of adding DGP to TTG antibodies, for the diagnosis of celiac disease (CeD) in the immunoglobulin A (IgA)-sufficient population.

**Methods:**

We included consecutive patients with suspected CeD who had both TTG and DGP serology performed simultaneously from 2017–2020 in Hamilton, Canada. Chart review was performed by 3 reviewers to extract data on biopsies, diagnosis of CeD and genetic HLA-DQ2/DQ8. CeD was defined as positive serology (either TTG and/or DGP) and villous atrophy in duodenal biopsies (≥Marsh-3a). A case was defined as an instance of TTG and DGP performed at a single timepoint. A single patient could have represented multiple cases if TTG and DGP were measured at multiple time points. Sensitivity, specificity, negative and positive predictive values were calculated, and ROC curves were generated. Diagnostic odds ratios (DOR) assessed the performance of each serological strategy compared to duodenal biopsies.

**Results:**

There were 580 patients constituting 823 cases that met inclusion criteria, of whom 441 had CeD. IgA-deficient patients (n=100) were excluded. Of the 723 cases remaining, 337 (214 adult;123 pediatric) had serology performed at the time of CeD diagnosis. TTG increased the odds of CeD diagnosis compared with DGP, Diagnostic Odds Ratio (DOR)=53.22 (95% CI 22.63–119.80) vs DOR=21.28 (95% CI 10.67–42.46). The addition of DGP to TTG did not increase the odds of CeD diagnosis [DGP+TTG DOR=51.39 (95% CI 19.36–135.61) vs TTG alone DOR=53.22 (95% CI 22.63–119.80)]. There were 37 discordant cases where only one of either TTG or DGP was positive. HLA-DQ2/DQ8 were absent in 2/9 cases with isolated increased DGP. Among the discordant cases, TTG outperformed DGP (DOR TTG= 4.29; 95% CI 1.09–16.83 vs DOR DGP=0.23; 95% CI 0.06–0.92).

**Conclusions:**

In the IgA-sufficient population, the addition of DGP to TTG testing does not increase the diagnostic accuracy of CeD serologic screening. This has implications in health-care costs as false positive results prompt further investigations. Given these findings, larger prospective studies should be completed prior to adding DGP antibodies to routine TTG serology.

**Funding Agencies:**

None

